# Effects of stimbiotic supplementation on gut health, immune response, and intestinal microbiota in weaned piglets challenged with *E. coli*

**DOI:** 10.3389/fvets.2023.1187002

**Published:** 2023-07-19

**Authors:** Dongcheol Song, Jihwan Lee, Woogi Kwak, Hanjin Oh, Seyeon Chang, Jaewoo An, Hyunah Cho, Sehyun Park, Kyeongho Jeon, Jinho Cho

**Affiliations:** ^1^Department of Animal Science, Chungbuk National University, Cheongju, Republic of Korea; ^2^Department of Poultry Science, University of Georgia (UGA), Athens, GA, United States

**Keywords:** stimbiotic, immune response, gut health, *E. coli*, intestinal microbiota

## Abstract

In order to make piglet diets more effective, it is necessary to investigate effective methods for breaking down xylan in cereal. The objective of this study was to determine the effects of dietary stimbiotic (STB) supplementation on growth performance, intestinal morphology, immune response and intestinal microbiota in weaned piglets. A total of 24 (Duroc × Yorkshire × Landrace) weaned pigs (initial body weight of 8.01 ± 0.38 kg and 28 ± 3 d old), were assigned to 4 treatments with 6 replicates per treatment. Pigs were housed in individual pens for 17 days, including 5 days adaption period and 12 days after the first *Escherichia coli (E. coli)* challenge. The experiment was conducted in a 2 × 2 factorial arrangement of treatments consisting of two levels of challenge (challenge and non-challenge) and two levels of STB (0 and 0.5 g/kg diet). Supplementations of STB 0.5 g/kg improved the gain to feed ratio (G:F) (*P* < 0.05) in piglets challenged with shiga toxigenic *E. coli* (STEC). STB supplementation decreased (*P* < 0.05) white blood cells, neutrophils, lymphocytes, and expression levels of tumor necrosis factor-alpha and interleukin-6. Supplementation of STB improved (*P* < 0.05) the lymphocytes and neutrophils in piglets challenged with STEC on 12 dpi. Supplementation of STB also improved (*P* < 0.05) the villus height to-crypt depth ratio of ileum in piglets challenged with STEC. Supplementation of STB increased (*P* < 0.05) the expression levels of claudin-1 of ileum. In genus level, supplementation of STB increased (*P* < 0.001) the abundance of *Prevotella* compared to non-supplementation of STB groups in pre-inoculation period. Also, supplementation of STB decreased (*P* < 0.05) the abundance of *Faecalibacterium* and *Eubacterium_coprostanoligenes*_group compared to non-supplementation of STB groups in post-inoculation period. In phylum level, supplementation of STB increased (*P* < 0.05) the abundance of *Desulfobacterota* and *Fibrobacterota* in pre-inoculation period. *E. coli* challenge increased the abundance of *Fibrobacterota* compared to non-challenged group in post-inoculation period. In conclusion, these findings indicated that STB supplementation could alleviate a decrease of the performance, immune response, and inflammatory response in piglets induced by the STEC challenge.

## Introduction

Commonly, grains in feed contain variable amounts of non-starch polysaccharides (NSP), which can reduce absorption, decrease digestibility of nutrients, and increase digesta viscosity in the small intestines (1). Xylan, the most abundant fiber source in pig diets, is resistant to digestion by endogenous digestive enzymes (2). Additionally, xylan can result in gut leakage and inflammation by viscous digesta (3). To make piglet diets more effective, it is necessary to investigate effective methods for breaking down xylan into cereal.

Xylanase (XYL), a carbohydrase that can degrade NSP, has been used in the diets of monogastric animals to mitigate the growth performance (4). Zheng et al. (5) reported that xylanase supplementation reduced digesta viscosity and improved nutrient digestibility. According to Petry and Patience (6), supplementation of XYL improved growth performance and villus height in weaned piglets challenged with *Escherichia coli (E. coli)*. Similarly, a mixture of XYL and protease supplementation mitigated the proliferation of coliforms in the ceca and improved the growth performance of the broilers (7).

Xylo-oligosaccharides (XOS) are derived from the hydrolysis of xylan and are made up of xylose monomers bonded together with β-(1, 4) linkages (8). In addition, XOS are considered prebiotics which are non-digestible dietary components that selectively fermented in the intestine (9). Such selective fermentation can change the activity of gut microbiome and promote short-chain fatty acid (SCFA) production (10).

Stimbiotic (STB), a complex of XYL and XOS, means “non-digestible but fermentable additive that obviously activates fiber-degrading microbiota to improve fiber fermentability at an insufficient amount” (11). Hence, STB has multi-function that reduces the antinutritive effects of NSP in feed and stimulates the microbiota to produce more SCFA (12). It may be possible to increase the fermentability of NSP by the supplementation of STB.

In the weaning period, piglets face new environments and experience intestinal morphological changes because of solid diet feeding (13). Post-weaning diarrhea (PWD) caused by these stress factors can lead to changes in gastrointestinal microbiology and immunology (14). Weaning stress has a negative impact on reduced feed intake, poor growth performance, and disease susceptibility (15). Especially, *E. coli* is the main cause of diarrhea, and gut microbiome is associated with diarrhea (16). For an experiment to be successful, it is crucial to develop a model that mimics an outbreak of PWD in a commercial setting. According to our previous studies (11, 14), we determined the optimal dosage of *E. coli*. Our previous study showed that supplementation of STB 0.5 g/kg and 1 g/kg improved gut health compared with non-supplementation of STB (11). However, supplementation of STB 0.5 g/kg showed a higher improvement in immune response such as pro-inflammatory cytokines compared with supplementation of STB 1 g/kg. However, we conducted the experiment and the effects of dietary STB supplementation of 0.5 g/kg on growth performance, intestinal health, and immune response in weaned piglets challenged with the PWD infection model. Therefore, we hypothesized that (1) experimental induction of PWD could increase the damage of intestinal mucosa and inflammatory response and (2) supplementation of STB 0.5 g/kg could reduce the antinutritive effects of NSP and provide beneficial bacteria which improve gut health and immunity. To test these hypotheses, we induced inflammation and gut damage through oral inoculation of *E. coli* and then investigated the effects of STB supplementation on gut health.

## Materials and methods

### Ethics approval and consent to participate

The protocol for this study was reviewed and approved by the Institutional Animal Care and Use Committee of Chungbuk National University, Cheongju, Korea (approval no. CBNUA-1697-22-01).

### Bacterial strains, culture, and challenges

Shiga toxin-producing *E. coli* F18 was provided in stock form. The F18 *E. coli* expressed heat-labile toxin (LT) and Shiga toxin type 2e (stx2e). In total, 10 μl of thawed *E. coli* stock was inoculated into 10 ml of nutrient broth and cultured at 37°C for 24 h and then subcultured. Thereafter, the subcultured *E. coli* was smeared on MacConkey agar to confirm the bacterial enumeration. A final concentration of 1.2 × 10^10^ CFU/ml was used in this study.

### Animals, experimental design, and diets

A total of 24 (Duroc × Yorkshire × Landrace) weaned pigs (initial body weight of 8.01 ± 0.38 kg and 28 ± 3 days old) were assigned to 4 treatments with 6 replicates per treatment. Pigs were housed in individual pens for 17 days, including 5 days of adaptation period and 12 days after the first *E. coli* challenge (d 0). The experiment was conducted in a 2 × 2 factorial arrangement of treatments consisting of two levels of challenge (challenge and non-challenge) and two levels of STB (0 and 0.5 g/kg diet). Corn and soybean meal basal diets were formulated to meet or exceed the nutrient requirements for the weaned piglets as recommended by NRC (Table 1) (17). STB used in this study was obtained by a commercial company (Eugene-Bio, Suwon, South Korea). The pigs were fed daily at 8:30 and 17:00 h and had *ad libitum* access to water. Feed residues were removed before the next meal and considered in the calculations. In the *E. coli* challenge treatments, all pigs were orally inoculated by dividing 10 ml of *E. coli* F18 for 3 consecutive days. Challenged piglets and non-challenged piglets were housed in a separate room. Strict biosecurity procedures were followed to avoid *E. coli* contamination of the non-challenged piglets.

**Table 1:** Compositions of basal diets (as-fed-basis).

**Items**	**Content**
**Ingredients, %**	
Corn	34.43
Extruded corn	15.00
Lactose	10.00
Dehulled soybean meal, 51% CP^a^	13.50
Soy protein concentrate, 65% CP^a^	10.00
Plasma powder	6.00
Whey	5.00
Soy oil	2.20
Monocalcium phosphate	1.26
Limestone	1.40
L-Lysine-HCl, 78%	0.06
DL-Methionine, 50%	0.15
Choline chloride, 25%	0.10
Vitamin premix^b^	0.25
Trace mineral premix^c^	0.25
Salt	0.40
Total	100.00
**Calculated value**	
ME, Kcal/kg	3433
CP, %	20.76
Lysine, %	1.35
Methionine, %	0.39
Ca	0.82
P	0.65
**Analyzed value**	
ME, kcal/kg	3512
CP, %	20.92

a CP, crude protein.

b Provided per kg of complete diet: vitamin A, 11,025 IU; vitamin D_3_, 1103 IU; vitamin E, 44 IU; vitamin K, 4.4 mg; riboflavin, 8.3 mg; niacin, 50 mg; thiamine, 4 mg; d-pantothenic, 29 mg; choline, 166 mg; and vitamin B_12_, 33 mg.

c Provided per kg of complete diet without Zinc: Cu (as CuSO_4_•5H_2_O), 12 mg; Mn (as MnO_2_), 8 mg; I (as KI), 0.28 mg; and Se (as Na_2_SeO_3_•5H_2_O), 0.15 mg.

### Growth performance

All piglets were weighed every week during the experimental period, and feed consumption was recorded to calculate average daily gain (ADG), average daily feed intake (ADFI), and gain-to-feed ratio (G:F).

### Diarrhea scores

The diarrhea scores were individually recorded at 08:00 and 17:00 h by the same person during the entire experimental period. The diarrhea score was scored using a method used by Zhao et al. (18). The diarrhea scores were assigned as follows: 0, normal feces; 1, soft feces; 2, mild diarrhea; and 3, severe diarrhea.

### Nutrient digestibility

To estimate the digestibility, 0.2% chromium oxide (Cr_2_O_3_) was supplemented with diets as an indigestible marker. Pigs were fed diets mixed with chromium oxide for 4 consecutive days from days post-inoculation (DPI) 4 and 12, and fresh excreta samples were collected in that period. At the end of the experiment, fecal samples were stored at −20°C and dried at 70°C for 72 h and then ground to pass through a 1 mm screen. All analysis items (feed and fecal) were analyzed for DM and CP. The procedures utilized for the determination of dry matter (DM) and crude protein (CP) digestibility were conducted with the AOAC methods (19). Chromium was analyzed with an ultraviolet absorption spectrophotometer (UV-1201, Shimadzu, Kyoto, Japan). The digestibility was calculated using the following formula: digestibility (%) = [1–(Nf × Cd)/(Nd × Cf)] × 100, where Nf is the nutrient concentration in feces (% DM), Nd is the nutrient concentration in diet (% DM), Cd is the chromium concentration in diet (% DM), and Cf is the chromium concentration in feces (% DM).

### Blood profile

Blood samples were obtained from the jugular vein of 6 pigs, each treatment at dpi 0, dpi 2, dpi 4, dpi 7, and dpi 12. At the time of collection, blood samples were collected into vacuum tubes containing K_3_EDTA for CBC analysis and non-heparinized tubes for serum analysis, respectively. After collection, blood samples were centrifuged (3,000 × g for 15 min at 4°C). The white blood cells (WBC), basophils, neutrophils, and lymphocyte levels in the whole blood were measured using an automatic blood analyzer (ADVIA 120, Bayer, NY, USA).

### Morphological analysis of small intestine

At the end of the experiment (dpi 12), pigs were anesthetized with carbon dioxide gas after blood sampling and euthanized by exsanguination. Intestinal tissues of approximately 10 cm from the ileum (close to the ileocecal junction) were collected and fixed in 10% neutral buffered formalin (NBF; Sigma–Aldrich, St. Louis, MO, United States). After cutting the intestine sample, it was dehydrated and dealcoholized. The samples were, then, installed on slides, treated with paraffin, and stained with hematoxylin and eosin. Villus height (VH) and crypt depth (CD) were measured under the light microscope (OLYMPUS DP71, BX50F-3, Olympus Optical Co. Ltd., Tokyo, Japan). VH was determined by measuring the distance between the tip of the villi to the villus crypt junction, and CD was determined by measuring the distance between adjacent villi.

The hematoxylin-eosin-stained slides were also used for goblet cell counting. In crypts, goblet cells were counted in the five best-oriented crypts/intestinal tract, from crypt mouth to base (adjacent to submucosa). The number of goblet cells is expressed as the mean number per crypt per tract and mean number of goblet cells/100 μm of crypts (mean data of crypt length). This was determined in order to supply the number of cells/crypt (anatomo-functional unit), flanked by number of goblet cells/unit length of epithelium (linear density), that is more comparable with bibliographic data (Obj. 40X). The equation to determine the number of goblet cells/100 μm was: goblet cells/100 μm = number of goblet cells × 100/(crypt depth × 2) (20). In villi, goblet cells were counted in the five best-oriented villi/intestinal tract, from villus tip to base (adjacent to crypt mouth). The number of goblet cells is expressed as the mean number/villus per tract and the mean number of goblet cells/100 μm of villi epithelium (mean data of villus height).

### Measurements of pro-inflammatory cytokine and immunoglobulin

The inflammatory biomarkers such as interleukin-6 (IL-6) and tumor necrosis factor α (TNF*-*α) were measured using commercially available ELISA kits, according to the manufacturer's instructions (R&D Systems, Minneapolis, MN). Immunoglobulin G (IgG) and immunoglobulin A (IgA) levels were gauged using an automatic biochemistry blood analyzer (Hitachi 747; Hitachi, Tokyo, Japan).

### Expression of tight junction proteins

The intestinal sample stored at −80°C after sampling was homogenized and used for calprotectin and claudin-1 (CLDN-1) concentration analysis. The concentration of total protein was quantified using a Pierce BCA protein assay kit (#23225, Thermo Fisher Scientific, Waltham, MA, USA). After the homogenized intestinal sample was diluted to reach a working range of 20–2,000 μg/ml, the absorbance was measured at 562 nm. The total protein concentration was calculated as a standard curve and used to normalize the concentrations of calprotectin and CLDN-1. The relative protein expression of calprotectin and CLDN-1 was determined by using commercially available ELISA kits (Cat no. MBS707210, MBS025129; Mybiosource, San Diego, CA, USA). Homogenized intestinal samples were diluted to reach a working range of 0.312–20 ng/ml for calprotectin and 0.5–16 ng/ml for CLDN-1. Both absorbances were measured at 450 nm. The concentrations of calprotectin and CLDN-1 were calculated by the standard curve and described as ng/mg of protein.

### 16S metagenomic data analysis

Bacterial 16S rRNA sequencing data of the two different metagenomics sequencing methods were analyzed using QIIME2 next-generation microbiome bioinformatics pipeline for comparative metagenomics study. The samples were sent to Sanigen (Anyang, South Korea) for microbial sequencing using the 16s rRNA technique. All raw input data were transformed in the form of QIIME2 artifacts, which contain information about the data types and sources for the downstream processing. From raw sequence data, the amplicon sequence variants (ASVs) were obtained using the Divisive Amplicon Denoising Algorithm 2 (DADA2) within QIIME 2 plugin, which detected and corrected amplicon errors and filtered out the potential base error and chimeric sequences (21, 22). The relative classification frequency table represented differential abundance tests at specific taxonomic levels was created using collapse and feature-table within the QIIME2 plugins. The “diversity” QIIME2 plugin was used to estimate alpha diversity measurements and plots using the R bioinformatics packages. This microbial diversity analysis pipeline was designed to use the ASV table (a higher resolution analog than the traditional OTU table) of the ASV picking step as necessary input data. Analyzing the differences in species richness and evenness scores considering the sampling depth was measured using the observed OTUs and Chao1, Shannon, and Simpson indices. Each index estimates the V3-V4 hypervariable region of the bacterial 16S rRNA gene. In addition, a difference in the relative abundance was analyzed by comparing the average bacterial proportion and composition investigated in each taxonomic ranking. Additionally, according to the different amplicon regions, the bacterial classification accuracy was cross-checked by comparing the taxonomy matching rate of each ASV taxonomy and NCBI bacterial reference genome database at the phylum and genus levels.

### Statistical analysis

Statistical analyses and graph construction were performed using JMP Pro 16 (SAS Institute Inc., Cary, NC, United States) and GraphPad Prism (Version 9.1.0; GraphPad Software, San Diego, CA), respectively. Parametric data (growth performance, ileal morphology, blood profile, cytokine level, and TJ proteins) were submitted to two-way ANOVA using the Standard Least Squares model. The statistical model included the effect of the *E. coli* challenge (chal -, chal +), the effect of STB supplementation (0, 0.5 g/kg), and the interaction between *E. coli* and STB, and initial body weight at the start of the trial (d 0) was also included as a covariate. The richness and alpha diversity were calculated with raw counts based on Shannon estimators. For quantitative beta diversity measurement, each treatment group was placed as the control group, and treatment groups were compared by using PROC MIXED with Dunnett's *post-hoc* test. Non-parametric data (diarrhea score) were analyzed using contingency analysis to test the relationship between categorical variables (scores) and the different combinations tested in this study. A chi-square test was performed to determine if the different combinations had an effect on the categorical variables, repartition with significance accepted at *P* < 0.05.

## Results

### Growth performance

The effects of STB supplementation on the growth performance of piglets are presented in Table 2. There was an interaction (*P* < 0.05) between the supplementation of STB and *E. coli* challenge in BW, ADG, ADFI, and G:F. Piglets supplemented STB5 with *E. coli* challenge had higher BW on 7 dpi (*P* = 0.005) and final (*P* = 0.002) compared with piglets fed STB0 with *E. coli* challenge.

**Table 2:** Effects of stimbiotic supplementation on growth performance in pigs challenged with STEC.

**Items**	**-C**		+**C**		**SE^1^**	**C**		**STB**		***P*** **-value**		
	**0**	**0.05**	**0**	**0.05**		**-**	**+**	**0**	**0.05**	**C**	**STB**	**CxSTB**
**BW, kg**												
Initial (D-5)	8.01	8.03	8.00	8.01	0.170	8.02	8.00	8.01	8.02		0.938	
D0	8.28	8.23	8.23	8.37	0.157	8.25	8.30	8.25	8.30		0.762	
D7	10.12^a^	9.89^ab^	9.36^b^	10.09^a^	0.149	10.01	9.72	9.74	9.99	0.073	0.106	0.005
Final (D12)	11.13^a^	11.16^a^	10.35^b^	11.17^a^	0.108	11.14	10.76	10.74	11.16	0.002	0.001	0.002
**Pre**												
**d - 5 to 0**												
ADG, kg	0.05	0.04	0.05	0.07	0.013	0.05	0.06	0.05	0.06		0.592	
ADFI, kg	0.17	0.16	0.15	0.18	0.021	0.16	0.16	0.16	0.17		0.708	
G:F	0.31	0.25	0.26	0.37	0.047	0.28	0.32	0.29	0.31		0.620	
**Post**												
**d 0 to 7**												
ADG, kg	0.26^a^	0.24^a^	0.16^b^	0.25^a^	0.017	0.25	0.20	0.21	0.24	0.014	0.112	0.005
ADFI, kg	0.34^ab^	0.34^ab^	0.33^b^	0.35^a^	0.005	0.34	0.34	0.34	0.35	0.510	0.028	0.006
G:F	0.76^a^	0.70^a^	0.49^b^	0.70^a^	0.044	0.73	0.59	0.62	0.70	0.006	0.124	0.007
**d 7 to 12**												
ADG, kg	0.25	0.32	0.25	0.27	0.018	0.28	0.26	0.25	0.29	0.177	0.028	0.277
ADFI, kg	0.40	0.40	0.40	0.40	0.001	0.40	0.40	0.40	0.40	0.717	0.039	0.717
G:F	0.64	0.79	0.62	0.68	0.045	0.71	0.65	0.63	0.73	0.172	0.034	0.291
**d 0 to 12**												
ADG, kg	0.26^a^	0.27^a^	0.19^b^	0.25^a^	0.011	0.26	0.22	0.23	0.26	0.002	0.005	0.020
ADFI, kg	0.36^ab^	0.36^ab^	0.35^b^	0.37^a^	0.003	0.36	0.36	0.36	0.37	0.543	0.022	0.009
G:F	0.71^a^	0.73^a^	0.55^b^	0.69^a^	0.026	0.72	0.62	0.63	0.71	0.001	0.005	0.034

-C, non-challenge with STEC; +C, challenge with STEC; 0 and 0.05, supplementation of STB 0, 0.05%; BW, body weight; ADG, average daily gain; ADFI, average daily feed intake; G:F, gain-to-feed ratio; Pre, pre-inoculation period; Post, post-inoculation period.

l SE, standard error.

a, b Values within a row with different superscripts are significantly different.

### Incidence of diarrhea

**Figure 1:**
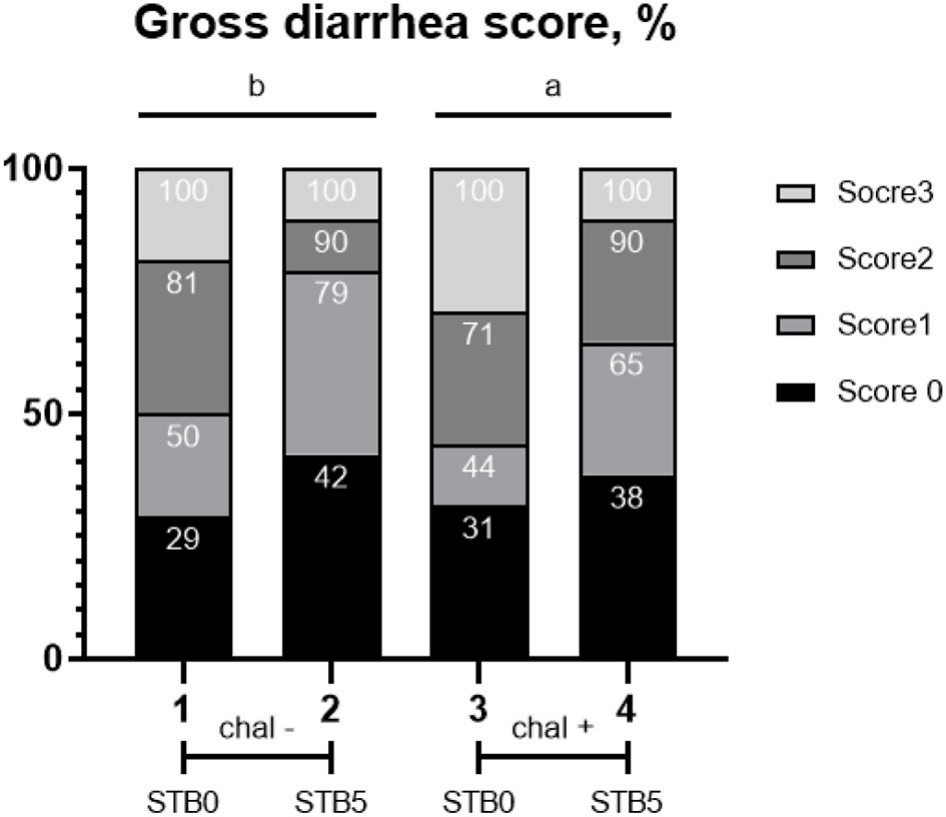
Effects of STB in weaned piglets challenged with *E. coli* on diarrhea score. χ2 = 19.571, *P* = 0.0208. Numbers inside the bar indicate the percentage of score out of total (100%) as shown in the legend. ^a,b^Mean scores followed by different superscripts in the bar graph indicate statistical significance by Student's *t*-test (*P* < 0.05).

An overview of the incidence of diarrhea is shown in Figure 1. After the *E. coli* challenge, there were differences in diarrhea scores from 1 to 7 dpi. *E. coli* challenge increased (*P* = 0.0004) the average diarrhea scores compared with the non-challenged group. Supplementation of STB5 decreased (*P* < 0.001) the average diarrhea scores compared with supplementation of STB0. There was an interaction between supplementation of STB and *E. coli* challenge in diarrhea score on days 0 to 12.

### Nutrient digestibility

*E. coli* challenge decreased the CP digestibility on 1w and 2w compared with the non-challenged groups (Table 3). There was an interaction between STB and *E. coli* challenge in digestibility on 2w. Piglets supplemented with STB5 with *E. coli* challenge had higher digestibility of CP, DM, and GE on 2w compared with piglets supplemented with STB0 with *E. coli* challenge.

**Table 3:** Effects of stimbiotic supplementation on digestibility in pigs challenged with STEC.

**Items, %**	**-C**		+**C**		**SE^1^**	**C**		**STB**		***P*** **-value**		
	**0**	**0.05**	**0**	**0.05**		**-**	**+**	**0**	**0.05**	**C**	**STB**	**C × STB**
**Post**												
**1 W**												
DM	89.92	89.62	89.96	89.81	0.308	89.77	89.89	89.94	89.72	0.705	0.486	0.803
CP	70.19	69.30	66.62	68.57	0.774	69.74	67.60	68.40	68.93	0.012	0.501	0.081
GE	71.13	70.78	69.94	70.39	1.276	70.96	70.17	70.54	70.58	0.543	0.970	0.761
**2 W**												
DM	90.81^a^	90.30^ab^	89.28^b^	91.05^a^	0.384	90.56	90.16	90.05	90.68	0.316	0.117	0.008
CP	71.40^a^	70.92^a^	65.65^b^	70.81^a^	1.319	71.16	68.23	68.53	70.86	0.018	0.052	0.022
GE	73.49^a^	74.10^a^	67.52^b^	74.86^a^	1.325	73.79	71.19	70.50	74.48	0.057	0.006	0.017

-C, non-challenge with STEC; +C, challenge with STEC; 0 and 0.05, supplementation of STB 0, 0.05%; Post, post-inoculation period.

l SE, standard error.

a, b Values within a row with different superscripts are significantly different.

### Blood profile

*E. coli* challenge increased (*P* < 0.05) WBC and neutrophils and decreased (*P* < 0.05) lymphocytes compared with the non-challenged group during the post-inoculation period (Table 4). The piglets fed STB5 had improved (*P* < 0.05) neutrophils and lymphocytes in 12 dpi compared with STB0. There was an interaction between STB and *E. coli* challenge in WBC, neutrophils, and lymphocytes. Piglets supplemented STB5 with *E. coli* challenge improved WBC (*P* = 0.024), neutrophils (*P* = 0.002), and lymphocytes (*P* = 0.002) compared with piglets fed STB0 with *E. coli* challenge on 12 dpi.

**Table 4:** Effects of stimbiotic supplementation on blood profile in pigs challenged with STEC.

**Items**	**-C**		+**C**		**SE^1^**	**C**		**STB**		***P*** **-value**		
	**0**	**0.05**	**0**	**0.05**		**-**	**+**	**0**	**0.05**	**C**	**STB**	**C × STB**
**D 0**												
WBC, 10^3^/μL	15.65	15.69	15.07	15.13	0.761	15.67	15.09	15.36	15.41		0.947	
Bas, %	0.07	0.10	0.07	0.03	0.026	0.08	0.50	0.07	0.07		1.000	
Neu, %	48.20	50.73	53.00	47.00	3.406	49.47	50.00	50.60	48.87		0.616	
Lym, %	46.53	43.67	40.60	46.97	3.466	45.10	43.78	43.57	45.32		0.619	
N:L	1.04	1.19	1.47	1.07	0.170	1.12	1.27	1.26	1.13		0.472	
**Post**												
**D 2**												
WBC, 10^3^/μL	16.78	16.53	25.58	25.07	1.486	16.53	25.32	21.18	20.80	< 0.001	0.803	0.931
Bas, %	0.03	0.03	0.07	0.07	0.021	0.03	0.07	0.05	0.05	0.130	1.000	1.000
Neu, %	50.53	51.57	55.00	57.17	2.125	51.05	56.08	52.77	54.37	0.028	0.460	0.793
Lym, %	41.27	44.97	35.23	33.13	2.502	43.12	34.18	38.25	39.05	0.002	0.753	0.260
N:L	1.31	1.15	1.57	1.82	0.138	1.23	1.69	1.44	1.48	0.003	0.757	0.152
**D 4**												
WBC, 10^3^/μL	15.89	15.69	28.25	26.11	1.187	15.78	27.18	22.07	20.90	< 0.001	0.336	0.421
Bas, %	0.07	0.07	0.07	0.07	0.028	0.07	0.07	0.07	0.07	1.000	1.000	1.000
Neu, %	47.67	48.13	62.53	55.73	3.651	47.90	59.13	55.10	51.93	0.006	0.396	0.332
Lym, %	45.37	46.60	30.47	39.97	3.509	45.98	35.22	37.92	43.28	0.006	0.142	0.253
N:L	1.14	1.11	2.27	1.47	0.255	1.12	1.87	1.70	1.29	0.009	0.123	0.145
**D 7**												
WBC, 10^3^/μL	15.74	15.38	26.13	20.02	1.922	15.56	23.07	20.94	17.70	0.001	0.108	0.150
Bas, %	0.10	0.07	0.07	0.10	0.030	0.08	0.08	0.08	0.08	1.000	1.0000	0.277
Neu, %	44.93	42.17	51.83	47.40	4.024	43.55	49.62	48.38	44.78	0.147	0.382	0.838
Lym, %	45.70	48.90	40.67	43.73	3.268	47.30	47.20	43.18	46.32	0.134	0.349	0.984
N:L	1.08	0.90	1.30	1.13	0.153	0.99	1.22	1.20	1.02	0.154	0.274	0.990
**D 12**												
WBC, 10^3^/μL	15.76^b^	16.48^b^	21.38^a^	17.59^b^	0.925	16.12	19.48	18.57	17.03	0.002	0.112	0.024
Bas, %	0.10	0.10	0.07	0.07	0.024	0.10	0.07	0.08	0.08	0.173	1.000	1.000
Neu, %	40.87^b^	40.80^b^	47.23^a^	40.70^b^	0.906	40.83	43.97	44.05	40.75	0.003	0.002	0.002
Lym, %	53.13^a^	52.47^a^	47.10^b^	53.00^a^	0.903	52.80	50.05	50.12	52.73	0.006	0.009	0.002
N:L	0.77^b^	0.78^b^	1.01^a^	0.77^b^	0.033	0.78	0.89	0.89	0.77	0.002	0.002	0.001

-C, non-challenge with STEC; +C, challenge with STEC; 0 and 0.05, supplementation of STB 0, 0.05%; WBC, white blood cell; Bas, basophils; Neu, neutrophils; Lym, lymphocytes; N:L, neutrophils to lymphocytes ratio; Post, post-inoculation period.

l SE, standard error.

a, b Values within a row with different superscripts are significantly different.

### Measurements of pro-inflammatory cytokine

*E. coli* challenge increased (*P* < 0.05) TNF-α and IL-6 and decreased (*P* < 0.05) IgG compared with the non-challenged group during the post-inoculation period (Table 5). Supplementation of STB5 improved (*P* < 0.05) TNF-α on 2 and 12 dpi compared with STB0. In addition, supplementation of STB5 improved (*P* < 0.05) IL-6 on 4, 7, and 12 dpi compared with STB0. There was an interaction between STB and *E. coli* challenge in TNF- α and IL-6. Piglets supplemented with STB5 with *E. coli* challenge improved (*P* < 0.05) IL-6 on 4, 7, and 12 dpi compared with piglets fed STB0 with *E. coli* challenge. In addition, piglets supplemented STB5 with *E. coli* challenge improved (*P* < 0.05) TNF-α compared with piglets fed STB0 with *E. coli* challenge on 12 dpi.

**Table 5:** Effects of stimbiotic supplementation on cytokine in pigs challenged with STEC.

**Items**	**-C**		+**C**		**SE^1^**	**C**		**STB**		***P*** **-value**		
	**0**	**0.05**	**0**	**0.05**		**-**	**+**	**0**	**0.05**	**C**	**STB**	**C × STB**
**Pre**												
**D 0**												
TNF-α, pg/mL	70.65	67.22	66.91	64.14	4.372	68.94	65.52	68.78	65.68		0.486	
IL-6, pg/mL	1178.78	1182.68	1177.88	1174.74	56.355	1180.73	1176.31	1178.33	1178.71		0.995	
IgG, mg/dL	227.00	224.00	223.33	223.00	6.206	225.50	223.17	225.17	223.50		0.791	
IgA, mg/dL	1.00	1.33	1.17	1.17	0.158	1.17	1.17	1.08	1.25		1.000	
**Post**												
**D 2**												
TNF-α, pg/mL	69.72	61.27	125.49	89.62	8.519	65.50	107.55	97.60	75.45	< 0.001	0.017	0.123
IL-6, pg/mL	1233.76	1246.47	2469.85	2064.38	146.923	1240.11	2267.11	1851.80	1655.42	< 0.001	0.196	0.170
IgG, mg/dL	213.67	219.33	163.00	173.00	11.349	216.50	168.00	188.33	196.17	< 0.001	0.498	0.851
IgA, mg/dL	1.67	1.67	1.33	1.00	0.316	1.67	1.17	1.50	1.33	0.130	0.604	0.604
**D 4**												
TNF-α, pg/mL	68.32	80.22	143.95	118.13	21.948	74.27	131.04	106.13	99.17	0.018	0.754	0.400
IL-6, pg/mL	1200.32	1064.56	2729.34	1983.21	102.468	1132.44	1356.28	1964.83	1523.89	< 0.001	< 0.001	0.007
IgG, mg/dL	205.00	209.00	138.00	153.33	5.054	207.00	145.67	171.50	181.17	< 0.001	0.071	0.276
IgA, mg/dL	1.33	1.67	1.33	1.33	0.279	1.50	1.33	1.33	1.50	0.557	0.557	0.557
**D 7**												
TNF-α, pg/mL	60.87	59.60	118.66	93.81	10.961	60.24	106.23	89.76	76.71	< 0.001	0.248	0.295
IL-6, pg/mL	1166.43^c^	1152.87^b^	2334.19^a^	1575.26^b^	52.438	1159.65	1954.73	1750.31	1364.07	< 0.001	< 0.001	< 0.001
IgG, mg/dL	200.00	211.33	170.33	193.33	10.515	205.67	181.83	185.17	202.33	0.035	0.118	0.585
IgA, mg/dL	1.33	1.67	1.33	1.33	0.279	1.50	1.33	1.33	1.50	0.557	0.557	0.557
**D 12**												
TNF-α, pg/mL	59.03^bc^	53.28^c^	100.90^a^	67.08^b^	2.598	56.16	83.99	79.97	60.18	< 0.001	< 0.001	< 0.001
IL-6, pg/mL	1040.77^b^	1036.96^b^	1826.10^a^	1075.99^b^	76.957	1038.87	1451.05	1433.43	1056.48	< 0.001	< 0.001	< 0.001
IgG, mg/dL	219.33	217.00	177.33	196.67	10.252	218.17	187.00	198.33	206.83	0.007	0.417	0.303
IgA, mg/dL	1.33	1.33	1.67	1.00	0.258	1.33	1.33	1.50	1.17	1.000	0.211	0.211

-C, non-challenge with STEC; +C, challenge with STEC; 0 and 0.05, supplementation of STB 0, 0.05%; TNF-α, tumor necrosis factor alpha; IL-6, interleukin-6; IgG, immunoglobulin G; IgA, immunoglobulin A; Pre, pre-inoculation period; Post, post-inoculation period.

l SE, standard error.

a, b, c Values within a row with different superscripts are significantly different.

### Morphological analysis of the small intestine

**Figure 2:**
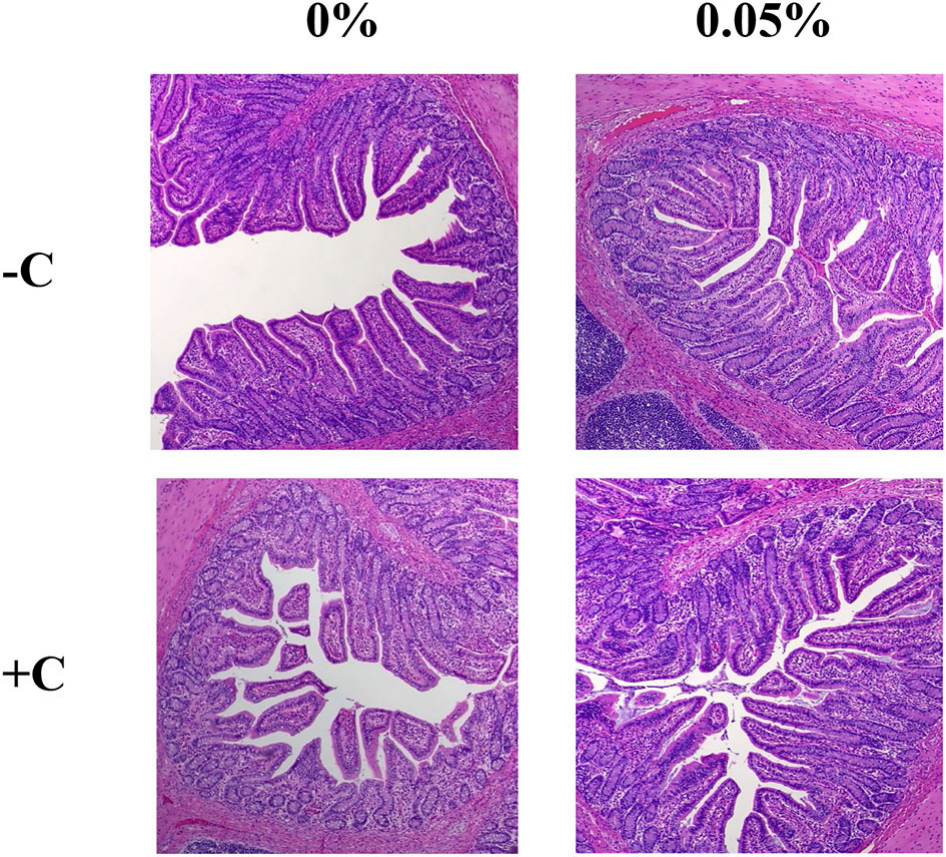
Histological analysis of the intestinal morphology of weaned piglets. Figures display the morphology of the ileum tissue from pigs in four dietary treatments.

There was no interaction between supplementation of STB and *E. coli* challenge in villus height, crypt depth, and height-to-depth ratio. *E. coli* challenge decreased (*P* = 0.021) villus height of the ileum (Figure 2, Table 6). Supplementation of STB increased (*P* > 0.05) VH and HDR. However, there was no difference between *E. coli* and supplementation of STB in CD. There was no interaction between supplementation of STB and *E. coli* challenge in counts of goblet cells. *E. coli* challenge increased (*P* = 0.005) the counts of goblet cells in the villus.

**Table 6:** Effects of stimbiotic supplementation on villus height and counts of goblet cell in pigs challenged with STEC.

**Items**	**-C**		+**C**		**SE^1^**	**C**		**STB**		***P*** **-value**		
	**0**	**0.05**	**0**	**0.05**		**-**	**+**	**0**	**0.05**	**C**	**STB**	**C × STB**
VH, μm	358.05	400.23	317.68	364.12	15.270	379.14	340.90	337.87	382.18	0.021	0.009	0.891
CD, μm	153.01	156.68	164.13	137.20	10.271	154.85	150.67	158.57	146.94	0.688	0.271	0.152
HDR	2.43	2.57	1.98	2.69	0.173	2.50	2.33	2.21	2.63	0.344	0.024	0.110
**Goblet cell**												
Villus	21.17	19.67	32.17	25.00	2.574	20.42	28.58	26.67	22.33	0.005	0.108	0.284
Crypt	19.83	19.33	21.00	20.83	0.908	19.58	20.91	20.42	20.08	0.158	0.718	0.856

-C, non-challenge with STEC; +C, challenge with STEC; 0 and 0.05, supplementation of STB 0, 0.05%; VH, villus height; CD, crypt depth; HDR, height to depth ratio.

l SE, standard error.

### Expression of tight junction proteins

**Figure 3:**
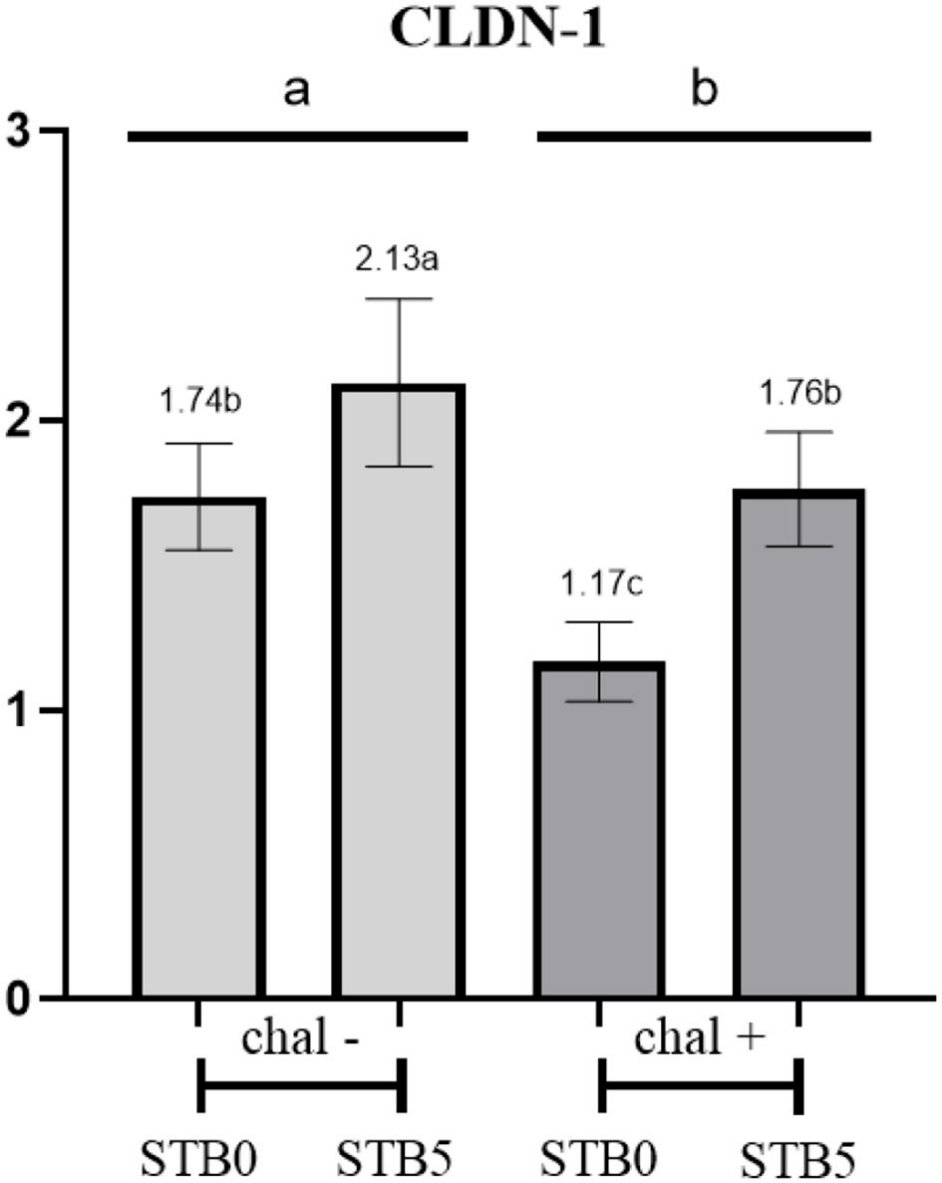
Effects of STB in weaned piglets challenged with *E. coli* on CLDN-1. ^a,b,c^Mean scores followed by different superscripts in the bar graph indicates statistical significance by Student's *t-*test (*P* < 0.05).

There was no interaction between the supplementation of STB and *E. coli* challenge in CLDN-1 and calprotectin (Figure 3). *E. coli* challenge downregulated (*P* < 0.05) the expression of CLDN-1 while supplementation of STB upregulated (*P* < 0.05) the expression of CLDN-1. In addition, the *E. coli* challenge upregulated (*P* < 0.001) the expression of calprotectin compared with the non-challenged group. However, supplementation of STB did not affect (*P* > 0.05) the expression of calprotectin.

### Alpha diversity of the fecal microbiome

No differences were observed in the alpha diversity parameters including Chao 1, Simpson, and Shannon indices on d 0 and dpi 12.

### Beta diversity of the fecal microbiome

**Figure 4:**
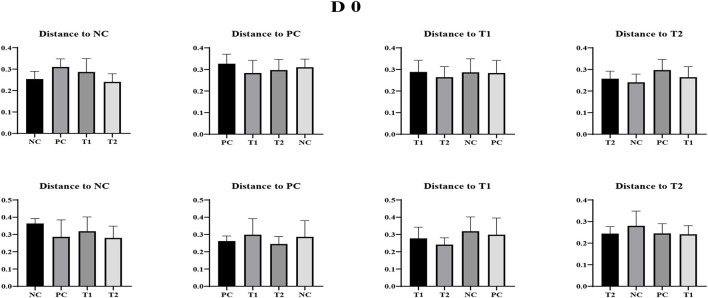
Weighted Unifrac measurement in negative control (NC): basal diet; positive control (PC): NC + *E. coli* challenge; treatment 1 (T1): NC + stimbiotic 0.5 g/kg; treatment 2 (T2): PC + stimbiotic 0.5 g/kg. Each treatment group was placed as the control group, and treatment groups were compared by using one-way PROC MIXED with Dunnett's *post-hoc* test.

**Figure 5:**
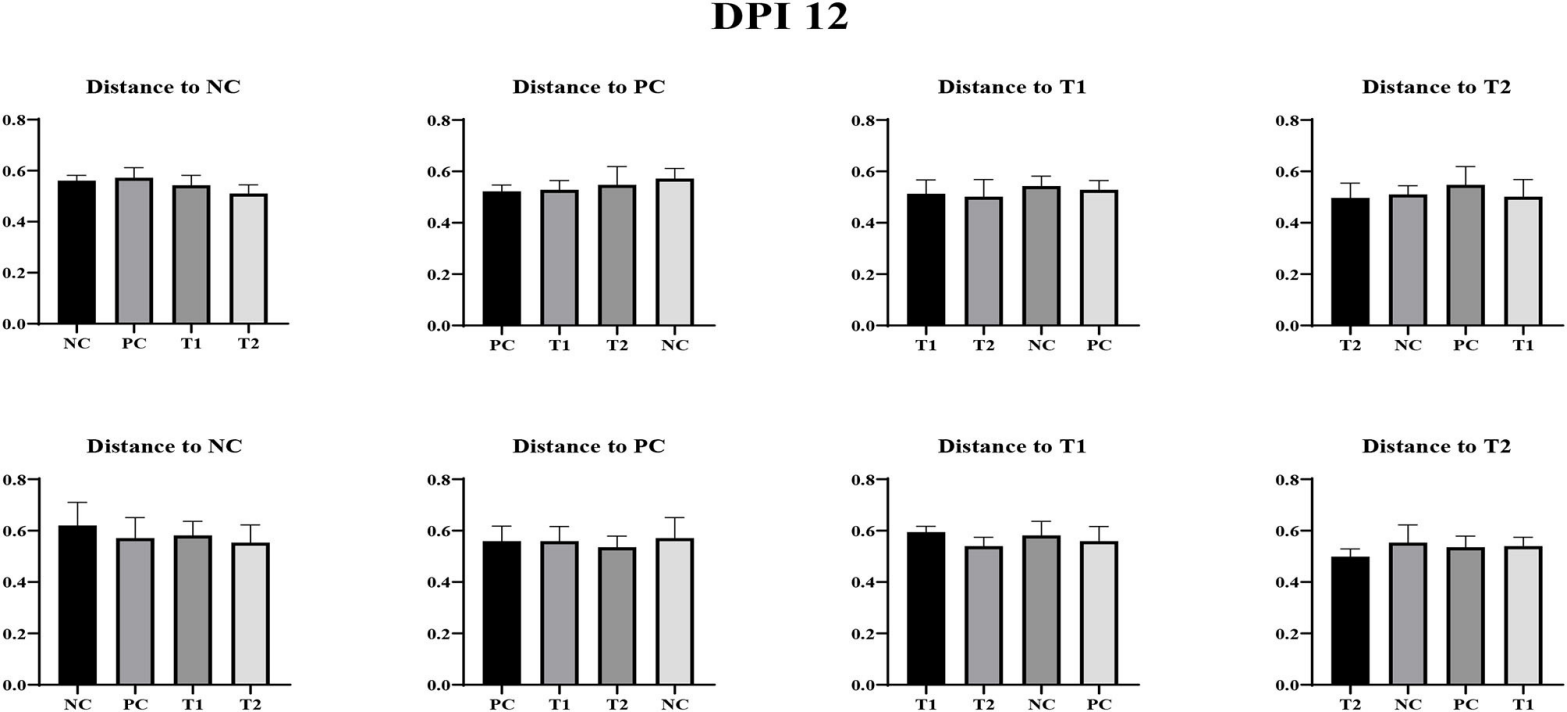
Unweighted unifrac measurement in negative control (NC): basal diet; positive control (PC): NC + *E. coli* challenge; treatment 1 (T1): NC + stimbiotic 0.5 g/kg; treatment 2 (T2): PC + stimbiotic 0.5 g/kg. Each treatment group was placed as the control group, and treatment groups were compared by using one-way PROC MIXED with Dunnett's *post-hoc* test.

**Figure 6:**
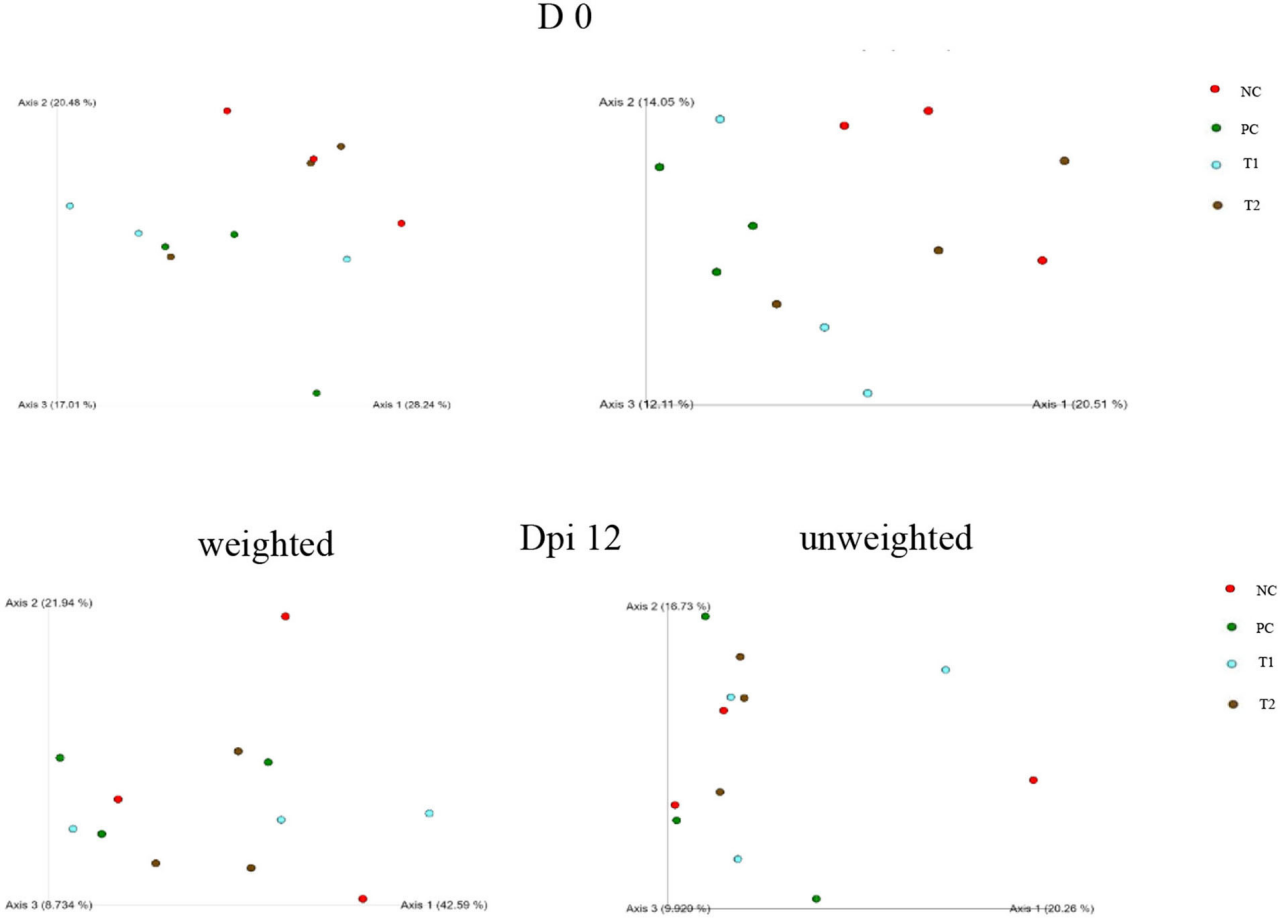
Visualized beta diversity indices including unweighted and weighted emperor in negative control (NC): basal diet; positive control (PC): NC + *E. coli* challenge; treatment 1 (T1): NC + stimbiotic 0.5 g/kg; treatment 2 (T2): PC + stimbiotic 0.5 g/kg.

No differences were observed in unweighted and weighted unifrac distance to each treatment both on d 0 and dpi 12 (*P* < 0.05; Figures 4–6).

### Relative abundance

**Figure 7:**
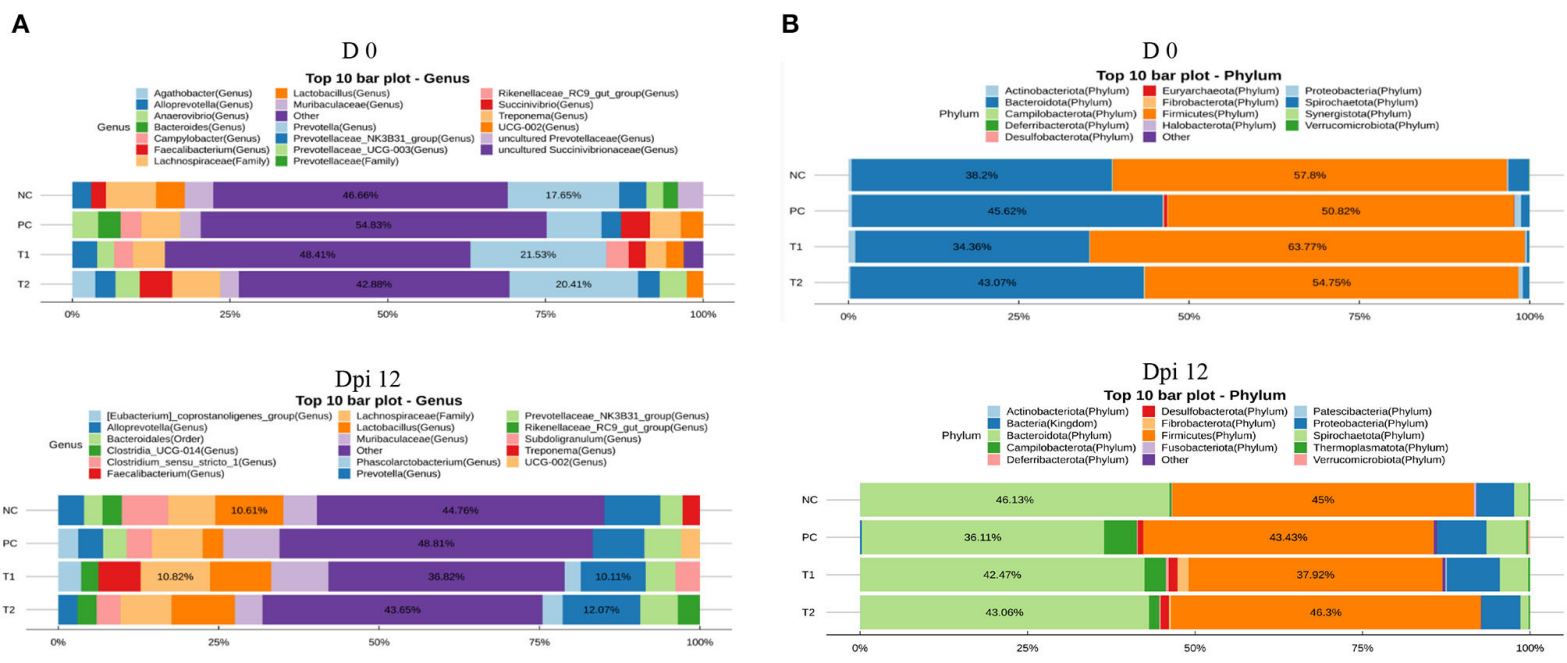
**(A)** 16S rRNA gene analysis revealed the relative abundance of fecal bacterial community structure at the genus level in piglets challenged with *E. coli* in negative control (NC): basal diet; positive control (PC): NC + *E. coli* challenge; treatment 1 (T1): NC + stimbiotic 0.5 g/kg; treatment 2 (T2): PC + stimbiotic 0.5 g/kg. **(B)** 16S rRNA gene analysis revealed the relative abundance of fecal bacterial community structure at the phylum level in piglets challenged with *E. coli* in negative control (NC): basal diet; positive control (PC): NC + *E. coli* challenge; treatment 1 (T1): NC + stimbiotic 0.5 g/kg; treatment 2 (T2): PC + stimbiotic 0.5 g/kg.

At the genus level, supplementation of STB increased (*P* < 0.001) the abundance of *Prevotella* compared with non-supplemented STB groups in the pre-inoculation period (Table 7; Figure 7). In addition, supplementation of STB decreased (*P* < 0.05) the abundance of the *Faecalibacterium* and *Eubacterium_coprostanoligenes*_group compared with the non-supplemented STB groups in the post-inoculation period. *E. coli* challenge decreased (*P* < 0.05) the abundance of *Clostridium_sensu_stricto_*1 and *Faecalibacterium* compared with the non-challenged groups in the post-inoculation period. There was an interaction between the supplementation of STB and *E. coli* challenge in the abundance of *Muribaculaceae* and *Faecalibacterium* in the post-inoculation period. Piglets supplemented STB5 with *E. coli* challenge decreased (*P* < 0.05) the abundance of *Muribaculaceae* and *Faecalibacterium* compared with piglets fed STB0 with *E. coli* challenge.

**Table 7:** Relative abundance of fecal microbiota at the genus level in pigs challenged with STEC on d 0 and 12 dpi and fed diets supplemented with stimbiotic.

**Items, %**	**-C**		+**C**		**SE^1^**	**C**		**STB**		***P*** **-value**		
	**0**	**0.05**	**0**	**0.05**		**-**	**+**	**0**	**0.05**	**C**	**STB**	**C × STB**
**Pre (d 0)**												
Prevotella	17.62	21.40	8.56	20.79	2.576	19.51	14.68	13.09	21.10		0.006	
Lachnospiraceae	8.05	5.09	6.29	7.72	1.403	6.57	7.00	7.17	6.41		0.593	
Anaerovibrio	2.16	2.67	4.13	3.65	1.017	2.41	3.89	3.14	3.16		0.985	
Muribaculaceae	4.61	1.96	3.25	2.94	1.034	3.29	3.10	3.93	2.45		0.469	
Alloprevotella	3.08	3.96	2.17	3.08	0.758	3.52	2.62	2.62	3.52		0.253	
Prevotellaceae_NK3B31_group	4.40	1.52	2.94	3.66	1.061	2.96	3.30	3.67	2.59		0.320	
Treponema	1.49	3.22	4.88	1.09	1.086	2.36	2.98	3.18	2.16		0.355	
Prevotellaceae_UCG-003	2.52	1.62	2.31	4.29	0.607	2.07	3.30	2.41	2.95		0.386	
Succinivibrio	1.06	2.65	4.47	1.68	0.820	1.85	3.08	2.77	2.16		0.471	
Rikenellaceae_RC9_gut_group	1.44	3.54	2.69	2.48	0.464	2.49	2.59	2.07	3.01		0.055	
Rest	53.57	52.37	58.32	48.62	1.808	52.97	53.47	55.94	50.49		0.007	
**Post (12 dpi)**												
Prevotella	8.50	8.28	10.08	11.95	1.502	8.39	11.01	9.29	10.11	0.096	0.588	0.496
Lachnospiraceae	7.43	7.79	10.75	8.06	1.042	7.61	9.40	9.09	7.93	0.101	0.279	0.160
Lactobacillus	10.39	3.39	9.51	10.05	3.038	6.89	9.78	9.95	6.72	0.353	0.300	0.229
Muribaculaceae	5.12	8.69	8.96	4.30	1.640	6.91	6.63	7.04	6.50	0.087	0.744	0.021
Prevotellaceae_NK3B31_group	3.38	5.34	4.77	6.21	1.885	4.36	5.49	4.07	5.78	0.555	0.377	0.894
Clostridium_sensu_stricto_1	7.21	4.09	1.85	3.63	1.200	5.65	2.74	4.53	3.86	0.025	0.583	0.055
Alloprevotella	3.94	4.09	1.62	3.07	1.452	4.01	2.35	2.78	3.58	0.265	0.586	0.660
Faecalibacterium	1.91	1.52	6.55	2.16	0.900	1.72	4.35	4.23	1.84	0.008	0.015	0.038
Eubacterium_coprostanoligenes_group	2.56	3.07	3.52	2.13	0.460	2.81	2.83	3.04	2.60	0.974	0.035	0.052
Clostridia_UCG-014	3.01	2.15	2.74	2.89	0.865	2.58	2.81	2.87	2.52	0.790	0.687	0.569
Rest	46.56	51.58	39.66	45.55	4.486	49.07	42.60	43.11	48.56	0.165	0.238	0.924

-C, non-challenge with STEC; +C, challenge with STEC; 0 and 0.05, supplementation of STB 0, 0.05%.

l SE, standard error.

In the phylum level, supplementation of STB increased (*P* < 0.05) the abundance of *Desulfobacterota* and *Fibrobacterota* in the pre-inoculation period (Figure 7, Table 8). However, supplementation of STB decreased (*P* = 0.040) the abundance of *Fibrobacterota* compared with the non-supplemented group in the post-inoculation period. *E. coli* challenge increased the abundance of *Fibrobacterota* compared with the non-challenged group in the post-inoculation period. There was an interaction between the supplementation of STB and *E. coli* challenge in the abundance of *Fibrobacterota*. Piglets supplemented STB5 with *E. coli* challenge decreased (*P* = 0.010) the abundance of *Fibrobacterota* compared with piglets supplemented STB0 with *E. coli* challenge.

**Table 8:** Relative abundance of fecal microbiota at the phylum level in pigs challenged with STEC on d 0 and 12 dpi and fed diets supplemented with stimbiotic.

**Items, %**	**-C**		+**C**		**SE^1^**	**C**		**STB**		***P*** **-value**		
	**0**	**0.05**	**0**	**0.05**		**-**	**+**	**0**	**0.05**	**C**	**STB**	**C × STB**
**Pre (d 0)**												
Firmicutes	44.94	38.04	43.93	46.16	2.459	41.49	45.05	44.44	42.10		0.354	
Bacteroidota	46.48	42.40	35.49	43.68	2.993	44.44	39.59	40.99	43.04		0.500	
Proteobacteria	5.48	7.87	7.35	5.52	1.659	6.67	6.44	6.41	6.69		0.868	
Spirochaetota	2.01	4.22	5.87	1.21	1.161	3.12	3.54	3.94	2.72		0.306	
Campilobacterota	0.30	3.26	5.03	1.47	1.157	1.78	3.25	2.66	2.37		0.799	
Desulfobacterota	0.01	1.37	0.76	1.18	0.424	0.69	0.97	0.39	1.28		0.049	
Fibrobacterota	0.03	1.56	0.03	0.27	0.373	0.79	0.15	0.03	0.92		0.027	
Thermoplasmatota	0.21	0.28	0.34	0.17	0.046	0.24	0.26	0.28	0.22		0.248	
Deferribacterota	0.09	0.39	0.19	0.19	0.085	0.24	0.19	0.14	0.29		0.097	
Fusobacteriota	0.32	0.16	0.02	0.01	0.104	0.24	0.01	0.17	0.09		0.431	
Rest	0.12	0.44	0.98	0.12	0.128	0.28	0.55	0.55	0.28		0.049	
**Post (12 dpi)**												
Firmicutes	57.84	63.66	51.06	54.46	4.156	60.75	52.76	54.45	59.06	0.069	0.281	0.775
Bacteroidota	37.90	34.48	45.31	43.36	4.281	36.19	44.33	41.61	38.92	0.072	0.538	0.865
Spirochaetota	3.28	0.37	1.29	0.93	0.969	1.82	1.11	2.29	0.65	0.469	0.107	0.203
Actinobacteriota	0.47	0.99	0.47	0.28	0.251	0.73	0.37	0.47	0.63	0.172	0.522	0.180
Proteobacteria	0.17	0.29	1.04	0.70	0.328	0.23	0.87	0.61	0.49	0.064	0.729	0.487
Euryarchaeota	0.04	0.00	0.39	0.02	0.111	0.03	0.20	0.22	0.01	0.122	0.080	0.153
Campilobacterota	0.16	0.06	0.08	0.12	0.051	0.11	0.10	0.12	0.09	0.884	0.544	0.192
Desulfobacterota	0.00	0.07	0.16	0.08	0.050	0.03	0.12	0.08	0.08	0.088	0.915	0.166
Fibrobacterota	0.00	0.02	0.12	0.00	0.024	0.01	0.06	0.06	0.01	0.040	0.040	0.010
Synergistota	0.04	0.03	0.02	0.04	0.021	0.04	0.03	0.03	0.03	0.609	0.945	0.552
Rest	0.09	0.04	0.06	0.02	0.026	0.06	0.04	0.07	0.30	0.362	0.115	0.977

-C, non-challenge with STEC; +C, challenge with STEC; 0 and 0.05, supplementation of STB 0 and 0.05%.

l SE, standard error.

## Discussion

Weaned piglets are commonly affected by PWD, which decreases their growth performance and increases their mortality (23). *E. coli* F18 is a primary pathogen associated with PWD in weaned piglets (24). It is known that *E. coli* F18 attaches to a specific receptor on the epithelium of the pig's intestinal tract through their fimbriae (25). As a result of colonization of the gut, toxins are produced, causing diarrhea (26). In many research facilities, experimental induction of PWD has been used to test the efficacy of functional additives under commercial PWD conditions (27–29). Furthermore, these studies have evaluated the gut health and immune response in weaned piglets challenged with *E. coli*.

The results obtained from this study showed that supplementation of 0.5 g/kg of STB could mitigate growth performance, diarrhea rate, intestinal morphology, and index of inflammation of weaned piglets challenged with *E. coli* infection. These results were consistent with other types of research, indicating an improved immune response after supplementation of STB (11, 30).

In the current study, F18 *E. coli* infection was accomplished in agreement with our previous studies using an *E. coli* challenge model in weaned piglets (9, 12). According to Kim et al. (31), heat-labile toxin and Shiga toxin induce gut permeability and inflammation, which, in turn, increase cytokines.

The integrity of the intestinal barrier is strictly regulated by Tight junction (TJ) proteins including CLDN, occludin, and zonula occludin which are continuously threatened by pro-inflammatory stimuli (32). Especially, CLDN-1, a representative TJ protein in mammals, enhances gut barriers to stop the loss of electrolytes (33). As a marker of neutrophilic inflammation in the gut, calprotectin concentrations are associated with the histological activity of inflammatory bowel disease (34). In the present study, supplementation of STB5 decreased the gross diarrhea incidence in *E. coli*-challenged piglets. This result is similar to those of previous studies showing that supplementing carbohydrase and XOS could alleviate diarrhea incidence in pigs (35, 36). It would be possible that increased expression of CLDN-1 in ileal mucosa fortified the intestinal barrier function in pigs fed STB infected with *E. coli*. TNF*-*α is a crucial pro-inflammatory cytokine in reaction to an infection by bacteria which is produced by neutrophils (31). IL-6 is also a pro-inflammatory cytokine expressed widely across vertebrates and plays multiple physiological roles involved in inflammation (37). TNF*-*α and IL-6 are served as biological indicators of intestinal inflammation in pigs (34, 38). Our observation indicated that supplementation of STB decreased the concentration of TNF*-*α and IL-6 in piglets infected with *E. coli*. Similar research reported that feeding STB improved TJ proteins between epithelial cells and reduced the production of pro-inflammatory cytokines such as TNF*-*α and IL-6 (39). Serum IgG and IgA are known to play an important role in humoral immunity.

The immune system depends on WBC, which includes neutrophils, lymphocytes, basophils, monocytes, and eosinophils, to control infections in the body, and the count of WBC is considered a marker of infection (40). The primary line of protection against bacterial infection is provided by neutrophils, and lymphocytes provide particular cellular and humoral immune responses (41). In the present study, *E. coli* infection increased counts of WBC and neutrophils, but supplementation of STB decreased the count of WBC and neutrophils in piglets infected with *E. coli*. In addition, the ratio of neutrophils to lymphocytes as a biomarker of inflammation was increased by *E. coli* infection. Similarly, the previous study reported that supplementation of mannan-oligosaccharide (MOS) decreased the neutrophils in broilers (42).

VH and CD are markers of enterocyte proliferation and villus damage (43). Shorter VH and deeper CD may indicate the presence of toxins (44). The villus contains enterocytes, goblet cells, and enteroendocrine cells that line the space, and the crypt contains undifferentiated cells and a subset of differentiated secretory cells (5). In our current study, *E. coli* infection decreased the VH, but supplementation of STB increased the VH, VH:CD, and goblet cells in the villi of piglets infected with *E. coli*. It has been reported that supplementation of MOS increased the villus height in weaned piglets (45). However, our results showed that supplementation of STB did not affect ileal CD. According to Luise et al. (46), XYL supplementation did not affect jejunal CD in weaned piglets which are genetically susceptible to ETEC. Zhang et al. (47) reported that the morphology of intestinal villi is tightly associated with the absorption of nutrients. Consistent with the results of the morphology of the small intestine, the digestibility of CP was also decreased by *E. coli* infection. There was an interaction effect between STB and *E. coli* infection. These findings support that supplementation of STB might mitigate inflammation and improve CP digestibility in *E. coli*-infected piglets.

Pathogenic challenges impair pig intestinal integrity by disturbing intestine microbial balance (48, 49). At the phylum level, *Fibrobacterota* is known for degrading lignocellulosic materials in the gut (47). Our study indicated that supplementation of STB increased the relative abundance of *Fibrobacterota* in the post-inoculation period. *Desulfobacterota* is associated with inflammation and increased relative abundance of *Desulfobacterota*, reducing VH and epithelial cells and downregulating the expression of TJ proteins (50). In the current study, the relative abundance of *Desulfobacterota* increased after *E. coli* infection. Similar research indicated that the abundance of *Desulfobacterota* is increased in broilers challenged with *C.perfrigens* (51).

At the genus level, *Prevotella* is associated with the production of acetate and butyrate in the small intestine (52). In the current study, supplementation of STB increased the relative abundance of *Prevotella*. Similar research indicated that the fermentation of xylan leads to the increased production of short-chain fatty acids such as butyrate (53).

## Conclusion

The results of this study support the hypothesis that supplementation of STB is capable of alleviating the growth performance and intestinal morphology, immune response, and gut microbiota in weaned piglets infected with *E. coli*. Our results supported that STB supplementation might increase the fermentability of NSP and reduce the antinutritive effects of NSP. Therefore, STB could be used as an antidiarrheal growth stimulator in weaned piglets.

## Data availability statement

The datasets presented in this study can be found in online repositories. The names of the repository/repositories and accession number(s) can be found below: https://www.ncbi.nlm.nih.gov/, SUB12953593.

## Ethics statement

The animal study was reviewed and approved by Institutional Animal Care and Use Committee of Chungbuk National University.

## Author contributions

DS, WK, and JL conducted the experiment and wrote the manuscript. HO, SC, JA, HC, SP, and KJ helped to conduct animal trial and laboratory work and helped to revise the manuscript. JC was the principal investigator and wrote the last version of the manuscript. All authors read and approved the final manuscript.

## References

[1] Lærke H Arent S Dalsgaard S Bach Knudsen K. Effect of xylanases on ileal viscosity, intestinal fiber modification, and apparent ileal fiber and nutrient digestibility of rye and wheat in growing pigs. J Anim Sci. (2015) 93:4323–35. 10.2527/jas.2015-9096 26440332

[2] Wang W Zheng D Zhang Z Ye H Cao Q Zhang C. Efficacy of combination of endo-xylanase and xylan-debranching enzymes in improving cereal bran utilization in piglet diet. Animal Bioscience. (2022) 35:1733. 10.5713/ab.21.0534 35798031 PMC9659467

[3] Baker JT Duarte ME Holanda DM Kim SW. Friend or Foe? Impacts of dietary xylans, xylooligosaccharides, and xylanases on intestinal health and growth performance of monogastric animals. Animals. (2021) 11:609. 10.3390/ani11030609 33652614 PMC7996850

[4] Cozannet P Kidd MT Neto RM Geraert P-A. Next-generation non-starch polysaccharide-degrading, multi-carbohydrase complex rich in xylanase and arabinofuranosidase to enhance broiler feed digestibility. Poult Sci. (2017) 96:2743–50. 10.3382/ps/pex084 28431149

[5] Zheng L Duarte ME Sevarolli Loftus A Kim SW. Intestinal health of pigs upon weaning: Challenges and nutritional intervention. Front Vet Sci. (2021) 8:628258. 10.3389/fvets.2021.628258 33644153 PMC7906973

[6] Petry AL Patience JF. Xylanase supplementation in corn-based swine diets: a review with emphasis on potential mechanisms of action. J Animal Sci. (2020) 98:skaa318. 10.1093/jas/skaa318 32970148 PMC7759750

[7] Barekatain MR Antipatis C Rodgers N Walkden-Brown SW Iji PA Choct M. Evaluation of high dietary inclusion of distillers dried grains with solubles and supplementation of protease and xylanase in the diets of broiler chickens under necrotic enteritis challenge. Poult Sci. (2013) 92:1579–94. 10.3382/ps.2012-02786 23687155

[8] Pang J Zhou X Ye H Wu Y Wang Z Lu D. The high level of xylooligosaccharides improves growth performance in weaned piglets by increasing antioxidant activity, enhancing immune function, and modulating gut microbiota. Front Nutr. (2021) 3:8. 10.3389/fnut.2021.764556 34938759 PMC8685398

[9] Wang X Xiao K Yu C Wang L Liang T Zhu H. Xylooligosaccharide attenuates lipopolysaccharide-induced intestinal injury in piglets via suppressing inflammation and modulating cecal microbial communities. Anim Nutr. (2021) 7:609–20. 10.1016/j.aninu.2020.11.008 34377847 PMC8326603

[10] Tang S Chen Y Deng F Yan X Zhong R Meng Q. Xylooligosaccharide-mediated gut microbiota enhances gut barrier and modulates gut immunity associated with alterations of biological processes in a pig model. Carbohydr Polym. (2022) 294:119776. 10.1016/j.carbpol.2022.119776 35868753

[11] Song D Lee J Kwak W Song M Oh H Kim Y. Stimbiotic supplementation alleviates poor performance and gut integrity in weaned piglets induced by challenge with *E. coli*. Animals. (2022) 12:1799. 10.3390/ani12141799 35883346 PMC9312148

[12] Morgan NK Gomes GA Kim JC. Comparing the efficacy of stimbiotic and a combination of xylanase and beta-glucanase, in broilers fed wheat-barley based diets with high or low AME. Poult Sci. (2021) 100:101383. 10.1016/j.psj.2021.101383 34438325 PMC8383100

[13] Oh HJ Kim MH Song MH Lee JH Kim YJ Chang SY. Effects of replacing medical zinc oxide with different ratios of inorganic. organic zinc or reducing crude protein diet with mixed feed additives in weaned piglet diets. Animals. (2021) 11:3132. 10.3390/ani11113132 34827863 PMC8614496

[14] Chang SY Song MH Lee JH Oh HJ Kim YJ An JW. Phytogenic feed additives alleviate pathogenic Escherichia coli-induced intestinal damage through improving barrier integrity and inhibiting inflammation in weaned pigs. J Anim Sci Biotechnol. (2022) 13:1–12. 10.1186/s40104-022-00750-y 36050784 PMC9438252

[15] Xiong X Tan B Song M Ji P Kim K Yin Y. Nutritional intervention for the intestinal development and health of weaned pigs. Front Vet Sci. (2019) 6:46. 10.3389/fvets.2019.00046 30847348 PMC6393345

[16] Peng S-S Li Y Chen Q Hu Q He Y Che L Jiang PP. Intestinal and mucosal microbiome response to oral challenge of enterotoxigenic escherichia coli in weaned pigs. Pathog. (2022) 11:160. 10.3390/pathogens11020160 35215105 PMC8879466

[17] National Research Council. 11th ed. Washington, DC: National Academy of Sciences (2012).

[18] Zhao PY Jung JH Kim IH. Effect of mannan oligosaccharides and fructan on growth performance, nutrient digestibility, blood profile, and diarrhea score in weanling pigs. J Anim Sci. (2012) 90:833–9. 10.2527/jas.2011-3921 21984718

[19] AOAC International. Official Methods of Analysis of AOAC int. 18th ed. Rev. 2nd ed. In:Hortwitz W and Latimer Jr GW, editors. Gaithersburg, MD: AOAC International (2007).

[20] Tugnoli B Piva A Sarli G Grilli E. Tributyrin differentially regulates inflammatory markers and modulates goblet cells number along the intestinal tract segments of weaning pigs. Livest Sci. (2020) 234:103996. 10.1016/j.livsci.2020.103996

[21] Bolyen E Rideout JR Dillon MR Bokulich NA Abnet CC Al-Ghalith GA. Reproducible, interactive, scalable, and extensible microbiome data science using QIIME 2. Nat Biotechnol. (2019) 37:852–7. 10.1038/s41587-019-0209-9 31341288 PMC7015180

[22] Callahan BJ McMurdie PJ Rosen MJ Han AW Johnson AJA Holmes SP. DADA2. High-resolution sample inference from Illumina amplicon data. Nat Methods. (2016) 13:581–3. 10.1038/nmeth.3869 27214047 PMC4927377

[23] Almeida JAS Liu Y Song M Lee JJ Gaskins HR Maddox CW. Escherichia coli challenge and one type of smectite alter intestinal barrier of pigs. J Anim Sci Biotechnol. (2013) 4:1–8. 10.1186/2049-1891-4-52 24359581 PMC3897994

[24] Luise D Spinelli E Correa F Salvarani C Bosi P Trevisi P. Effects of *E. coli* bivalent vaccine and of host genetic susceptibility to *E coli* on the growth performance and faecal microbial profile of weaned pigs. Livest Sci. (2020) 241:104247. 10.1016/j.livsci.2020.104247

[25] Coddens A Diswall M Ångström J Breimer ME Goddeeris B Cox E. Recognition of blood group ABH type 1 determinants by the FedF adhesin of F18-fimbriated *Escherichia coli*. J Biol Chem. (2009) 284:9713–26. 10.1074/jbc.M807866200 19208633 PMC2665092

[26] Coddens A Loos M Vanrompay D Remon JP Cox E. Cranberry extract inhibits *in vitro* adhesion of F4 and F18+ Escherichia coli to pig intestinal epithelium and reduces *in vivo* excretion of pigs orally challenged with F18+ verotoxigenic *E. coli*. Vet Microbiol. (2017) 202:64–71. 10.1016/j.vetmic.2017.01.019 28161211

[27] Luise D Lauridsen C Bosi P Trevisi P. Methodology and application of Escherichia coli F4 and F18 encoding infection models in post-weaning pigs. J Anim Sci Biotechnol. (2019) 10:1–20. 10.1186/s40104-019-0352-7 31210932 PMC6567477

[28] Fairbrother JM Nadeau É Bélanger L Tremblay CL Tremblay D Brunelle M. Immunogenicity and protective efficacy of a single-dose live non-pathogenic Escherichia coli oral vaccine against F4-positive enterotoxigenic Escherichia coli challenge in pigs. Vaccine. (2017) 35:353–60. 10.1016/j.vaccine.2016.11.045 27916413

[29] Kim JC Heo JM Mullan BP Pluske JR. Efficacy of a reduced protein diet on clinical expression of post-weaning diarrhoea and life-time performance after experimental challenge with an enterotoxigenic strain of *Escherichia coli*. Anim Feed Sci Technol. (2011) 170:222–30. 10.1016/j.anifeedsci.2011.08.012

[30] Cho HM Gonzalez-Ortiz G Melo-Duran D Heo JM Cordero G Bedford MR. Stimbiotic supplementation improved performance and reduced inflammatory response via stimulating fiber fermenting microbiome in weaner pigs housed in a poor sanitary environment and fed an antibiotic-free low zinc oxide diet. PLoS ONE. (2020) 15:e0240264. 10.1371/journal.pone.0240264 33170861 PMC7654836

[31] Kim K Ehrlich A Perng V Chase JA Raybould H Li X. Algae-derived β-glucan enhanced gut health and immune responses of weaned pigs experimentally infected with a pathogenic *E. coli*. Anim Feed Sci Technol. (2019) 248:114–25. 10.1016/j.anifeedsci.2018.12.004

[32] Sudan S Zhan X Li J. A novel probiotic bacillus subtilis strain confers cytoprotection to host pig intestinal epithelial cells during enterotoxic escherichia coli infection. Microbiol Spectrum. (2022) 10:e01257–21. 10.1128/spectrum.01257-21 35736372 PMC9430607

[33] Splichal I Donovan SM Splichalova Z Neuzil Bunesova V Vlkova E Jenistova V. Colonization of germ-free piglets with commensal Lactobacillus amylovorus, Lactobacillus mucosae, and probiotic *E. coli* Nissle 1917 and their interference with *Salmonella Typhimurium*. Microorganisms. (2019) 7:273. 10.3390/microorganisms7080273 31434337 PMC6722580

[34] Boeckman JX Sprayberry S Korn AM Suchodolski JS Paulk C Genovese K. Effect of chronic and acute enterotoxigenic E. coli challenge on growth performance, intestinal inflammation, microbiome, and metabolome of weaned piglets. Sci Rep. (2022) 12:1–14. 10.1038/s41598-022-08446-z 35323827 PMC8943154

[35] Li Q Burrough ER Gabler NK Loving CL Sahin O Gould SA. A soluble and highly fermentable dietary fiber with carbohydrases improved gut barrier integrity markers and growth performance in F18 ETEC challenged pigs. J Anim Sci. (2019) 97:2139–53. 10.1093/jas/skz093 30888017 PMC6488326

[36] González-Solé F Solà-Oriol D Ramayo-Caldas Y Rodriguez-Prado M González Ortiz G Bedford MR. Supplementation of xylo-oligosaccharides to suckling piglets promotes the growth of fiber-degrading gut bacterial populations during the lactation and nursery periods. Sci Rep. (2022) 12:1–13. 10.1038/s41598-022-15963-4 35804098 PMC9270449

[37] Felcher CM Bogni ES Kordon EC. IL-6 cytokine family: a putative target for breast cancer prevention and treatment. Int J Mol Sci. (2022) 23:1809. 10.3390/ijms23031809 35163731 PMC8836921

[38] Sun Y Duarte ME Kim SW. Dietary inclusion of multispecies probiotics to reduce the severity of post-weaning diarrhea caused by Escherichia coli F18+ in pigs. Anim Nutr. (2021) 7:326–33. 10.1016/j.aninu.2020.08.012 34258420 PMC8245796

[39] Tiwari UP Fleming SA Rasheed MSA Jha R Dilger RN. The role of oligosaccharides and polysaccharides of xylan and mannan in gut health of monogastric animals. J Nutr Sci. (2020) 9:14. 10.1017/jns.2020.14 32595966 PMC7303790

[40] Hong J Ariyibi S Antony L Scaria J Dilberger-Lawson S Francis D Woyengo TA. Growth performance and gut health of Escherichia coli–challenged weaned pigs fed canola meal-containing diet. J Anim Sci. (2021) 99:skab196. 10.1093/jas/skab054.156 34159354 PMC8349558

[41] Kim K He Y Jinno C Kovanda L Li X Song M Liu Y. Trace amounts of antibiotic exacerbated diarrhea and systemic inflammation of weaned pigs infected with a pathogenic *Escherichia coli*. J. Anim Sci. (2021) 99:skab073. 10.1093/jas/skab073 33693730 PMC8480179

[42] Ghazalah AA El-Manylawi MAF Motawe HFA Khattab M Youssef Y. Growth performance, nutrient digestibility, biochemical properties, hematological traits, and intestinal histopathology of broiler chicks fed mannan oligosaccharides. World's Vet J. (2021) 11:621–33. 10.54203/scil.2021.wvj79

[43] Duarte ME Kim SW. Significance of mucosa-associated microbiota and its impacts on intestinal health of pigs challenged with F18+ *E. col*i. Pathog. (2022) 11:589. 10.3390/pathogens11050589 35631110 PMC9145386

[44] Csernus B Czeglédi L. Physiological, antimicrobial, intestine morphological, and immunological effects of fructooligosaccharides in pigs. Archives Animal Breeding. (2020) 63:325. 10.5194/aab-63-325-2020 32964103 PMC7500070

[45] Agazzi A Perricone V Omodei Zorini F Sandrini S Mariani E Jiang X-R. Dietary mannan oligosaccharides modulate gut inflammatory response and improve duodenal villi height in post-weaning piglets improving feed efficiency. Animals. (2020) 10:1283. 10.3390/ani10081283 32731342 PMC7459834

[46] Luise D Motta V Boudry C Salvarani C Correa F Mazzoni M. The supplementation of a corn/barley-based diet with bacterial xylanase did not prevent diarrhoea of ETEC susceptible piglets, but favoured the persistence of Lactobacillus reuteri in the gut. Livest Sci. (2020) 240:104161. 10.1016/j.livsci.2020.104161

[47] Zhang Z Huang B Wang Y Zhan Y Zhu M Wang C. Dynamic alterations in the donkey fecal bacteria community and metabolome characteristics during gestation. Front Microbiol. (2022) 13:561. 10.3389/fmicb.2022.927561 36060774 PMC9434018

[48] Duarte ME Tyus J Kim SW. Synbiotic effects of enzyme and probiotics on intestinal health and growth of newly weaned pigs challenged with enterotoxigenic F18+ Escherichia coli. Front Vet Sci. (2020) 7:573. 10.3389/fvets.2020.00573 33033721 PMC7509054

[49] Li Q Peng X Burrough ER Sahin O Gould SA Gabler NK. Dietary soluble and insoluble fiber with or without enzymes altered the intestinal microbiota in weaned pigs challenged with enterotoxigenic *E. coli* F18. Front Microbiol. (2020) 11:1110. 10.3389/fmicb.2020.01110 32536908 PMC7267687

[50] Tang Y Zhang X Wang Y Guo Y Zhu P Li G. Dietary ellagic acid ameliorated Clostridium perfringens-induced subclinical necrotic enteritis in broilers via regulating inflammation and cecal microbiota. J Anim Sci Biotechnol. (2022) 13:1–18. 10.1186/s40104-022-00694-3 35436978 PMC9016943

[51] Stanley D Hughes RJ Moore RJ. Microbiota of the chicken gastrointestinal tract: influence on health, productivity and disease. Appl Microbiol Biotechnol. (2014) 98:4301–10. 10.1007/s00253-014-5646-2 24643736

[52] Ivarsson E Roos S Liu H Lindberg J. Fermentable non-starch polysaccharides increases the abundance of Bacteroides–Prevotella–Porphyromonas in ileal microbial community of growing pigs. Animal. (2014) 8:1777–87. 10.1017/S1751731114001827 25046106

[53] Tiwari UP Singh AK Jha R. Fermentation characteristics of resistant starch, arabinoxylan, and β-glucan and their effects on the gut microbial ecology of pigs: a review. Anim Nutr. (2019) 5:217–26. 10.1016/j.aninu.2019.04.003 31528722 PMC6737498

